# Alpha-1 Antitrypsin Blood Levels as Indicator for the Efficacy of Cancer Treatment

**DOI:** 10.4021/wjon663e

**Published:** 2013-05-06

**Authors:** Zeyad J. El-Akawi, Aymen M. Abu-awad, Nabil A. Khouri

**Affiliations:** a Department of Physiology and Biochemistry, Faculty of Medicine, Jordan University of Science and Technology, Irbid 22110, Jordan; b Princess Iman Center of Research and Laboratory Sciences, King Hussein Medical Center, Royal Medical Services, Amman, Jordan; c Department of Anatomy, Faculty of Medicine, Jordan University of Science and Technology, Irbid 22110, Jordan

**Keywords:** Alpha-1 antitrypsin, Cancer, Treatment

## Abstract

**Background:**

Alpha-1 antitrypsin (α_1_-AT) is a member of the serine protease inhibitors (serpins) family. Liver cells are the major source of synthesis and secretion of (α_1_-AT) into the blood. Moreover, it has been demonstrated that α_1_-AT is expressed and secreted by many types of malignant cells. Studies have indicated that serum levels of (α_1_-AT) increase in a good number of malignant diseases. In addition, a significant correlation between serum levels and cancer stage has also been reported. In this work we aimed to test how α_1_-AT levels behave at the third week after treatment with chemotherapy.

**Methods:**

The α_1_-AT blood levels were measured using commercially available radial immunodiffusion kit (Kent Laboratory Inc, Bellinham, Washigton) following manufacturer instructions.

**Results:**

The α_1_-AT blood levels were significantly decreased after treatment compared with those before the treatment started. The mean difference (before - after) treatment was 127.82 and 137.37 mg/dL with 95% CI of difference 109.06 - 146.57 and 116.08 - 158.65 mg/dL in lung and prostate cancer respectively. When we compared these levels according to the stage of cancer, we found that the mean difference (before - after) treatment was also highly significant as indicated by P-value and the 95% CI of these differences.

**Conclusion:**

Obtained data strongly indicate the value of testing α_1_-AT blood levels as one of the important indicators for the efficacy of cancer treatment.

## Introduction

Alpha-1 antitrypsin (α_1_-AT) is a serum glycoprotein which synthesized mainly in human liver cells and macrophages. It is one of a serpins family which plays a central role in controlling tissue degradation through its inhibitory effect on neutrophile elastase and other serine proteases including; trypsin, chymotrypsin, cathepsin G, plasmin, thrombin, tissue kallikrein and activated factor X (FXa). These proteins constitute the third major protein component of blood plasma after albumin and the immunoglobulins [1-3]. It was demonstrated that many types of tumor cells are capable of expression and secretion of α_1_-AT [4-6]. In addition, It has been shown that α_1_-AT serum levels get elevated in a number of inflammatory diseases and different types of malignancies such as hepatocellular carcinoma, multiple myeloma, pancreatic carcinoma, prostatic carcinoma, primary carcinoma of the lung, cervical carcinoma, gastric cancer, laryngeal carcinoma, nasopharyngeal carcinoma, breast cancer and colorectal carcinoma [7-13]. These elevated levels were found to be correlated with the cancer staging as demonstrated in a good number of investigations [8, 14-17]. Considering the above facts concerning α_1_-AT and its relation to cancer, we decided to test how α_1_-AT blood levels behave during cancer treatment with chemotherapy and the correlation between the change in these levels and the treatment outcome.

## Material and Methods

In order to test for how α_1_-AT blood levels behave during the course of cancer treatment with chemotherapy, one hundred newly diagnosed untreated male cancer patients who are admitted to King Hussein Medical Center (K.H.M.C.) and Albashir Hospital in Amman were included in this study. The group of selected patients was consisting of fifty patients, mean age was 54 (35 - 70) years, with non-small cell lung cancer and the other fifty patients, mean age was 61 (50 - 70) years, with prostate cancer. Non-small cell lung cancer patients were classified based on the stage of the disease with the following distribution: 13 patients with stage I, 14 patients with stage II, 14 patients with stage III, and 9 patients with stage IV. The other fifty patients with prostate cancer were distributed based on the cancer staging as follows: 11 patients with stage A, 13 patients with stage B, 12 patients with stage C and 14 patients with stage D. Cancer staging were performed by specialist physicians. Venous blood samples were obtained and immediately processed to be used for the measurement of alpha-1 antitrypsin using commercially available radial immunodiffusion kit (Kent Laboratory Inc, Bellinham, Washigton) following manufacturer instructions. Alpha-1 AT blood levels were measured at two different time points, one before patients start their chemotherapy course and the other on the third week after chemotherapy course was started. A software package (Statistical Package for the Social Science, SPSS version 17) was used for calculations and statistical analysis. Statistical analysis was completed using paired t-test. A P-value of less than 0.05 (P < 0.05) was considered statistically significant.

## Results

Data obtained in this work demonstrated that α_1_-AT blood levels were significantly decreased on the third week after treatment with chemotherapy in all cancer patients included in this study except one patient with lung cancer. The summary of the obtained results are shown in the tables. Table 1 shows that the mean difference (before - after) treatment for α_1_-AT blood levels is highly significant in lung and prostate cancer as indicated by 95% confidence interval (CI) and the P-values. Table 2 and 3 are also demonstrate that the mean difference (before - after) treatment for α_1_-AT blood levels in lung and prostate cancer patients according to the stage of the disease is highly significant with the widening of the range of difference when we go toward more advanced stages.

**Table 1:** Mean Difference (Before - After) Treatment of Alpha-1 Antitrypsin Blood Levels in mg/dL in Lung and Prostate Cancer Patients

	Lung cancer	Prostate cancer
Number of patients	50	50
Mean difference (before - after) treatment	127.82	137.37
SE (mean difference)	9.33	10.59
95% CI of difference	109.06, 146.57	116.08, 158.65
P-value using paired (t) test	0.000	0.000

SE: Standard Error; CI: Coefficient Interval.

**Table 2:** Mean Difference (Before - After) Treatment of Alpha-1 Antitrypsin Blood Levels in mg/dL in Lung Cancer Patients According to Stages

Stages	I	II	III	IV
Number of patients	13	14	14	9
Mean difference (before - after) treatment	67.71	138.44	132.70	190.52
SE (mean difference)	13.78	15.36	13.90	17.23
95% CI of difference	37.68, 97.73	105.25, 171.63	102.68, 162.72	150.79, 230.25
P-value using paired (t) test	0.000	0.000	0.000	0.000

SE: Standard Error; CI: Coefficient Interval.

**Table 3:** Mean Difference (Before - After) Treatment of Alpha-1 Antitrypsin Blood Levels in mg/dL in Prostate Cancer Patients According to Stages

Stages	A	B	C	D
Number of patients	11	13	12	14
Mean difference (before - after) treatment	75. 93	119.91	148.93	191.24
SE (mean difference)	11.31	14.40	16.01	22.94
95% CI of difference	50.73, 101.13	88.54, 151.13	113.71, 184.16	141.68, 240.79
P-value using paired (t) test	0.000	0.000	0.000	0.000

SE: Standard Error; CI: Coefficient Interval.

## Discussion

Alpha-1 antitrypsin (α_1_-AT) is a glycoprotein expressed and secreted by normal cells such as liver, macrophages, lung, gall bladder, pancreas and gastrointestinal tract. Moreover, it has also been demonstrated that α_1_-AT is expressed in malignant cells including lung, thyroid, pancreatic cancer and others [4-8, 18]. Studies have indicated that alpha-1 antitrypsin (α_1_-AT) blood levels were significantly increased in a good number of malignant diseases [7-13]. In addition, it has been demonstrated that this increment in α_1_-AT blood levels is significantly correlated with cancer staging [8, 14-17]. In a previous study on lung and prostate cancer we demonstrated a direct and significant correlation between the elevated levels of the serum α_1_-AT and the stage of cancer [8]. Considering the above stated facts, we can suggest that the major source of the increased α_1_-AT blood levels in cancer patients is the growing cancer cells. In this work we measured α_1_-AT blood levels in untreated cancer patients before and three weeks after the treatment with chemotherapy. We demonstrated that these levels were significantly decreased at the third week after treatment. As indicated in the accompanied tables the mean difference (before - after) treatment is highly significant in both lung and prostate cancer. This difference is also significant between stages within the same type of cancer. Calculating the percentage of this decrease we found that the decrease in α_1_-AT blood levels is about 28.4% in lung cancer and about 28.9% in prostate cancer patients. The percentage of decrement in lung cancer patients was ranged from 5% to 46% and from 11% to 57% in prostate cancer patients. When the percentages were calculated considering the cancer staging in both lung and prostate cancer patients the percentages of decrement were found to be higher in later stages compared with early stages and this mostly due to the fact that the starting α_1_-AT blood levels in late stages is much higher compared with early stages. Several reports have shown that high α_1_-AT blood levels in cancer cases are associated with cancer spreading and worse prognosis [4, 14, 15, 19-22]. Tahara et al demonstrated that the two-year survival rates, among one hundred twenty-six gastric carcinoma patients, clearly indicated that well-differentiated adenocarcinomas with α_1_-AT have worse prognosis than well-differentiated adenocarcinomas without α_1_-AT [20]. Higashiyama M et al, in a clinical follow-up study of the patients with lung cancer, particularly in stage I, showed that strongly α_1_-AT -positive cases have poor prognosis than weak-to-moderately α_1_-AT -positive or α_1_-AT -negative cases. Therefore, they concluded that α_1_-AT expression status in tumor cells of lung adenocarcinoma may be a biological marker of prognostic significance in regard to tumor growth [4]. In addition, Karashima S et al suggested that alpha-1 AT in colorectal carcinoma is related to the invasive and metastatic capacity and thus it may serve as a biological marker for prognosis of colorectal carcinomas at relatively early stages [22]. In spite of a good number of publications concerning the relationship between α_1_-AT and tumor progression and poor prognosis, no special attention was given to this protein to be used for the evaluation of the outcome of cancer treatment. The only published work where it has been suggested that α_1_-AT serum levels might be used for the assessment of the efficacy of the treatment was by Konnova LA et al 1983 [23]. Our data came to confirm this suggestion and to strongly conclude that the measurement of α_1_-AT blood levels during the course of cancer treatment has a valuable clinical significance as indicator for the efficacy of treatment.
